# Separating the forest from the palm trees: Individual variation in a presurgical language mapping task

**DOI:** 10.1016/j.nicl.2026.103943

**Published:** 2026-01-05

**Authors:** Natalie L. Voets, Oiwi Parker Jones, Mohamed L. Seghier, Puneet Plaha

**Affiliations:** a Oxford Centre for Integrative Neuroimaging, University of Oxford, Oxford, UK; b Dept of Neurosurgery, Oxford University Hospitals NHS Foundation Trust, Oxford, UK; c PNPL, Dept of Engineering Science, University of Oxford, Oxford, UK; d Biomedical Engineering & Biotechnology Department, Khalifa University, Abu Dhabi, United Arab Emirates; e Nuffield Dept of Surgical Sciences, University of Oxford, Oxford, UK

**Keywords:** fMRI, Language, Tumour, Presurgical, Semantic, Glioma

## Abstract

**Background:**

Selecting optimal tasks for language mapping in neurosurgical patients poses challenges that are exacerbated by mismatches in practice between presurgical and intraoperative evaluations. To help align practices, we evaluated a functional MRI version of a semantic association task increasingly used during intra-operative assessment of awake neurosurgery patients. Using a recently proposed consistency mapping approach, we characterise task fMRI activation reliability across individuals, visits, and scan cohorts.

**Methods:**

FMRI data were acquired during an adapted Pyramids and Palm Trees Task (PPTT) in 15 healthy controls and 54 pre-surgical patients with a glioma. A new implementation of threshold-weighted overlap mapping (TWOM) was used to evaluate: 1. inter-individual variability in task activations among individuals; 2. test–retest variability in controls scanned twice (16 ± weeks apart); 3. between-scanner reliability across two patient cohorts scanned on a 3 T Siemens Prisma (n = 27) or Verio (n = 24) scanner using standard (TR = 3 s, voxel size 3 × 3 × 3 mm) or advanced (TR = 0.93 s, voxel size 2x2x2 mm) fMRI acquisitions, respectively.

**Results:**

Task-related activations in the core language network were highly consistent between individuals and across test–retest sessions. Several brain regions showed variable activations, reflecting atypical language dominance (confirmed during neurosurgery), or differences in regional involvement during semantic processing.

**Conclusion:**

The PPTT engaged widespread brain networks including but not limited to regions implicated in semantic processing. Overlap mapping is a powerful way to visualise meaningful variations in neural processing at the individual level, supporting alignment of pre- and intra-operative mapping for any given task.

## Highlights

• We evaluated a fMRI semantic association task for presurgical language mapping.
• Threshold weighted overlap mapping shows activation reliability over people & time.
• Overlap mapping reveals meaningful individual variations in task fMRI activity.

## 1 Introduction

Preservation of language function is a primary concern during surgical treatment of intrinsic brain tumours, or ‘glioma’, in the language dominant hemisphere. Current practices vary widely in cognitive (Rofes et al., 2017, Sierpowska et al., 2022, De Witte and Mariën, 2013), imaging-based (Stopa et al., 2020, Thust et al., 2018) and intra-operative (Rofes et al., 2017, Mandonnet et al., 2017) assessment of language functions. Optimal approaches for language mapping and monitoring therefore continue to be refined.

Task-based functional MRI (fMRI) remains the most commonly used non-invasive approach for pre-operative language mapping because of the ease with which it can measure the whole brain at high spatial resolution (Ojemann, 1983). The success of fMRI to localise functions is often compared to intra-operative electrocortical stimulation mapping (ESM). ESM aims to spare essential tissue from surgical damage by identifying areas where electrical current − applied directly to the brain − causes a reproducible change in function (Ojemann, 1983). ESM offers a widely-accepted clinical standard for operative mapping of brain function (Duffau, 2015) but is not always possible, and has a small risk of triggering a seizure during surgery (Talacchi et al., 2013).

Because fMRI and intra-operative stimulation mapping are mechanistically different, they provide complementary information (Voets et al., 2025a, Voets et al., 2025b). Therefore, direct comparison should not be expected to reach 100 %. Nonetheless, one practical use of fMRI is to inform functional risks to patients prior to surgery and help the surgeon focus and target cortical ESM. Consequently, broad agreement between the techniques is desirable. However, in practice, important differences arise in how language processing is assessed between the fMRI and surgical settings, including the specific language tasks performed (Weng et al., 2018, Holloway et al., 2022). For example, the majority of clinical fMRI examinations involve word generation tasks (Manan et al., 2020, Benjamin et al., 2018, Black et al., 2017) while intraoperative language mapping relies largely on picture naming (Rofes et al., 2017, De Witte and Mariën, 2013, Papatzalas et al., 2022). These different tasks call on different combinations of language processes that recruit different brain networks (Voets et al., 2025a). Therefore, to facilitate comparisons between results of pre- and intra-operative language mapping – and, ultimately, maximise the surgical utility of fMRI − many authors have advocated for a closer alignment of pre- and intra-operative language mapping practices (De Witte and Mariën, 2013, Voets et al., 2025a, Weng et al., 2018, Giussani et al., 2010).

Despite significant potential advantages, standardising language mapping throughout the neurosurgical workflow faces several hurdles. One challenge is a paucity of fMRI data from neurosurgical populations for specific language tasks commonly used both by neuropsychology and intraoperatively. Optimally, task selection should consider what language process(es) are most essential to map in each patient, and the level of specificity needed (Voets et al., 2025a). However, neural substrates are currently better understood for some language processes than others. For example, brain regions engaged in orthographic processing are reasonably well defined based on imaging and lesion data (Yeatman and White, 2021, Voets et al., 2025c). Conversely, the network of areas essential for semantic processing remains contentious and under active investigation (Cocquyt et al., 2020, Lambon Ralph et al., 2017).

The application of ESM during awake brain surgery first uncovered the role of the ventral stream, notably the inferior fronto-occipital fasciculus (IFOF), in semantic processing (Duffau et al., 2005). Multiple groups have since highlighted the importance of mapping this structure and its associated functional network in surgical glioma patients (Aabedi et al., 2022, Voets et al., 2021, Bello et al., 2010, Martino et al., 2015, Tuncer et al., 2021, Collée et al., 2023). These seminal findings were based on semantic paraphasias during picture naming (Duffau et al., 2005, Bello et al., 2007). The classic Pyramids and Palm Trees Test (PPTT) (Howard and Patterson, 1992) was subsequently adopted to target semantic processing deficits pre-, intra- and post-operatively (Gatignol et al., 2004, Moritz-Gasser et al., 2013). Surveys indicate that ∼ 50 % of European glioma centres now use the PPTT task during awake surgery (Rofes et al., 2017), while wider (e.g., international and epilepsy surgery) uptake is rising (de Zwart and Ruis, 2024). Given this growing use in surgery, we implemented and evaluated a fMRI version of the PPTT as a step to facilitate standardised assessment of semantic processing throughout the patient pathway.

The PPTT has been previously adapted for functional neuroimaging studies to explore the neural substrates of semantic decision-making. Most of these studies were conducted in healthy volunteers using the original task stimuli (Ricci et al., 1999, Vandenberghe et al., 1996, Seghier et al., 2011), or adaptations to modulate semantic features (Zhang et al., 2023). Additional PPTT neuroimaging studies explored semantic processing following stroke (Price et al., 1999, Gold and Kertesz, 2000), in healthy ageing (Baciu et al., 2016), and in neurodegenerative conditions (Vandenbulcke et al., 2006, McGeown et al., 2009, Nelissen et al., 2011). One prior study assessed the reproducibility of task activations in healthy controls performing semantic decisions on written word triplets adapted from the PPTT. Chee and colleagues (Chee, 2006) assessed fMRI test–retest activations for different frequency words over 2 sessions, collected one week apart, and noted “good overall correspondence”. Despite its widespread neuroscience applications, the PPTT has not, to our knowledge, previously been evaluated for its reliability to map semantic processing networks in presurgical patients using fMRI.

Here, we used a recently proposed ‘threshold-weighted overlap mapping’ approach (TWOM) (Seghier and Price, 2016) to characterise variability of task-based fMRI activity across a prospective cohort of presurgical glioma patients and healthy controls performing the PPTT. Our aims were: 1) to quantify test–retest consistency of activations in healthy controls; 2) to assess the reliability of activation maps from patients scanned on different scanners using different fMRI sequences. For a subset of patients who underwent awake surgery, we related the location of intraoperative semantic processing errors to their fMRI- and diffusion tractography-defined language networks. The present study highlights unique insights that overlap mapping provides into individual variations in language processing, and provide user-friendly scripts that can easily be applied to statistical maps generated using any other fMRI tasks.

## 2 Materials & methods

### 2.1 Participants

**Fig. 1:**
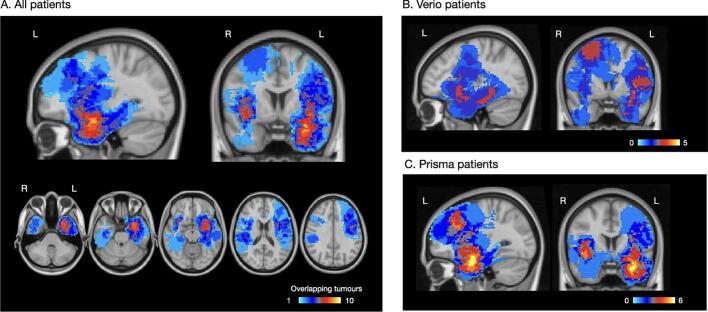
Tumour overlap map. Heat map showing the tumour locations across all patient participants (A) and separately for the tumour patients scanned on the Verio scanner (B) and Prisma scanner (C). Voxels with higher amounts of tumour overlap are indicated in warm (red-to-yellow) colours; less frequent tumour locations are shown in blue. (For interpretation of the references to colour in this figure legend, the reader is referred to the web version of this article.)

Patient participants were recruited prospectively through the neuro-oncology surgery service at the Oxford University Hospital (OUH) NHS Foundation Trust. Inclusion criteria were: aged 18 years and over; radiological diagnosis of a primary brain tumour; and undergoing neurosurgical treatment work-up. Patients were excluded if they had contraindications to MRI and prior surgery other than a diagnostic biopsy. Fifty-four patients completed pre-surgical research functional MRI. The fMRI data were subsequently excluded for 3 patients (see *FMRI results*). The patient characteristics for the remaining 51 patients (mean age 40.06 ± 12.78 years, range 19–70, 31 Male, 20 Female) are detailed in Table 1. Tumours affected predominantly the temporal, frontal and insular lobes (Fig. 1).

**Table 1:** Participant characteristics.

**Healthy controls**	
Age (mean, range in years)	37.7 ± 12.32
Sex (Male / Female)	8 / 7
Handedness (R / L / ambidextrous)	14 / 1

**Patient participants**	
Age (mean, range in years)	40.1 ± 12.8
Sex (Male / Female)	31 / 20
Handedness (R / L / ambidextrous)	43 / 6 / 2
*Tumour location* Language-dominant hemisphere Language non-dominant hemisphere	32 19
Histology (50 operated patients*) Astrocytoma WHO grade 2 Astrocytoma WHO grade 3 Astrocytoma WHO grade 4 Oligodendroglioma WHO grade 2 Oligodendroglioma WHO grade 3 Glioblastoma Other Infiltration zone diffuse glioma NOS, WHO grade 2 DNET, WHO grade 1	12 13 2 6 5 9 1 2

NOS: Not otherwise specified. DNET: Dysembryoplastic neuroepithelial tumour. WHO: World Health Organisation. * One patient refused surgery.

The control group consisted of 15 healthy participants (mean age 37.7 ± 12.32 years, range: 26–68, 8 Male, 7 Female), age-matched to the patient group (Mann-Whitney U = 334.5, p = 0.46). Exclusion criteria for healthy controls included contraindications to MRI and any current or prior neurological or psychiatric condition.

### 2.2 Ethics

This research study was approved by the Oxford-B Research Ethics Committee. The study was conducted in accordance with the principles of the Declaration of Helsinki. All participants gave informed written consent.

### 2.3 PPTT fMRI task design

**Fig. 2:**
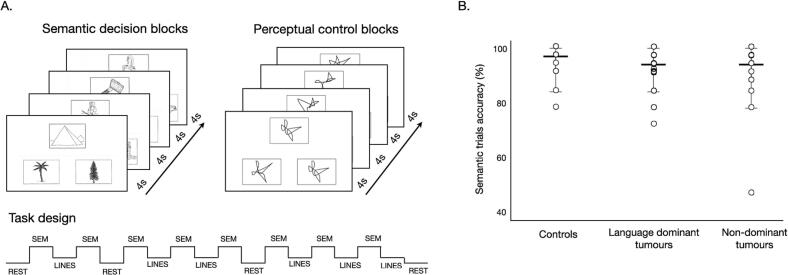
PPTT fMRI task design and performance accuracy. A. Details of our fMRI implementation of the Pyramids and Palm Trees Task, consisting of alternating blocks of semantic decisions on pictures (‘semantic matching’), perceptual matching of line drawings (‘line matching’), or resting fixation. Each active block contained 4 trials lasting 4 s each, followed by a short delay (3 s) before the start of the next block. The order of blocks, and the order of trials within each block, was held constant. B. Performance accuracy on semantic trials (%) was high across healthy controls and tumour patients, with no significant difference between them (independent-samples Mann-Whitney U tests, all p > 0.05).

During fMRI, semantic picture trials and line-matching trials were presented in alternating blocks (Fig. 2a) (Voets et al., 2021). During each block, 4 picture trials were presented for a duration of 4 s each, followed by a short 3 s inter-block delay. The semantic trials consisted of the original Pyramids and Palm Trees Test neuropsychological task stimuli (Howard and Patterson, 1992) (https://www.pearsonclinical.co.uk/). On each active task (i.e., ‘semantic’) trial, participants were simultaneously presented with a triplet of nameable picture items. Each triplet consisted of one target picture (e.g., of a pyramid) and two choice items (e.g., a pine tree and a palm tree). During control task blocks (‘line-matching’), participants were presented with 3 non-nameable black and white line drawings selected from a previously described dataset (Slotnick and Schacter, 2004). On every trial, participants indicated, using a MRI-compatible button response box, which of the two choice items was most *related* to the target (semantic blocks) or visually *identical* to the target (line-matching blocks). These line-matching blocks aimed to control for perceptual input, decision-making (choosing between items) and motor responses (button press) to better isolate the process of semantic association evoked during PPTT trials. Four resting fixation blocks of 14 s each were incorporated to verify the main effect for each condition (i.e., semantic > rest, lines > rest). In-scanner performance was recorded as percent of accurate responses across all semantic trials. Button responses were not captured for 1 control and 5 patients. To ensure understanding of the task, a practice session was performed with all participants using example test stimuli prior to the scan.

### 2.4 MRI acquisition

All MRI scans took place at the University of Oxford Centre for Integrative Neuroimaging. Functional MRI data were acquired using a blood oxygen dependent (BOLD) gradient echo, echo planar imaging sequence. To assess test–retest consistency, the 15 healthy controls were scanned twice on the same 3 T Siemens Prisma MRI scanner approximately 16 weeks apart (standard deviation 11.2, range 3.5 – 37 weeks). Patients were scanned once prior to surgery. Twenty-four patients were scanned on a 3 T Siemens Verio scanner with standard clinical-grade fMRI sequence parameters: TR = 3000 ms, TE = 28 ms, voxel resolution 3 x 3 x 3, flip angle: 90degrees, acquisition time 05:27. After decommissioning of the Verio, 30 new patients were scanned on a 3 T Siemens Prisma (the same as controls) with an advanced research-grade fMRI sequence: TR = 933 ms, TE = 33.4 ms, multiband acceleration factor 6, voxel resolution 2 x 2 x 2, flip angle: 64degrees, acquisition time 05:25. Excluding 3 motion-affected datasets (see *FMRI results*), the final Prisma patient cohort consisted of 27 patients.

Diffusion MRI data were acquired for tractography using an echo planar imaging sequence (TR = 2394 ms, TE = 79.40 ms, 1.75 × 1.75 × 1.75 mm voxel size, Multiband acceleration factor = 4). Diffusion data were acquired across 3 shells (b = 500 / 1,500 / 2500 s/mm^2^), in 12 / 48 / 48 directions, respectively, with 6b = 0 reference volumes. The sequence was acquired twice (04:50 per acquisition), using inverted phase-encode directions to correct for susceptibility weighted distortions and to improve the signal-to-noise ratio through averaging.

A high resolution 1 mm^3^ MPRAGE T1-weighted anatomical scan was acquired for co-registration and tumour delineation (see next section).

### 2.5 FMRI pre-processing and registration

Individual subject fMRI data were pre-processed using FMRIB’s software library (FSL, https://fsl.fmrib.ox.ac.uk/fsl/). Steps included motion correction between consecutive volumes, spatial smoothing at 5 mm full width at half maximum, high-pass filtering at 100 s and distortion correction using field maps (Smith et al., 2004). The fMRI data were registered to the individual’s high-resolution brain using boundary-based registration (Greve and Fischl, 2009) and then to the MNI standard-space template brain. For accurate alignment to the MNI template brain, tumour masks were first manually drawn on each patient’s T1 anatomical scan and visually verified by a consultant neuroradiologist. The anatomical scans were then nonlinearly registered to the MNI 2 mm template brain, excluding all voxels within the tumour mask.

### 2.6 Standard fMRI GLM analysis

The fMRI task data were analysed using a whole-brain General Linear Model (GLM) implemented in FSL’s FMRI Expert Analysis Tool, v6.0 (Woolrich et al., 2009). In brief, regions showing statistically higher BOLD signal when participants made semantic decisions relative to visual pairings (i.e. contrasting ‘semantic’ > ‘line-matching’) were identified at the individual subject level, modelling head motion parameters when indicated. In detail, the timings of the ‘semantic’ and ‘line matching’ task blocks were input to the GLM as separate ‘predicted responses’, each convolved with a haemodynamic response function. FEAT then estimated, for every brain voxel, the parameter values that best fit the fMRI data based on a linear combination of the predicted responses. Next, t-statistics were calculated to identify − from the voxel-wise parameter value maps and corresponding residual error maps − which voxels showed a greater response during ‘semantic’ than during ‘line matching’ blocks. The t-contrast maps were automatically converted to z-maps and corrected for multiple comparisons across the whole brain. The final activation (contrast) maps were generated using cluster statistics performed on all voxels at a cluster forming threshold of z = 3.1 and a corrected significance level of p < 0.05. The resulting individual first-level PPTT task activation maps (unthresholded contrast of parameter estimates) were visually inspected and combined in two ways. First, for standard group-level analyses, the individual activation maps were combined in a second-level fixed-effects GLM to obtain *shared variance* population maps, and to directly compare group-level activation maps (visit 1 vs visit 2 in healthy controls). A second, complementary analysis assessed *inter-subject variability* in task activations through overlap mapping (Seghier and Price, 2016, Ekert et al., 2021), described next.

### 2.7 FMRI task consistency mapping

To quantify variability in PPTT activation, we generated voxel-based threshold-weighted overlap maps (TWOM) from the individual-subject first-level task activation maps as previously described (Seghier and Price, 2016). The rationale for this approach is that research goals typically include identifying brain regions where participants show *shared signal* during a task (i.e. revealing the common task network), or identifying (e.g., disease-induced) *differences* at the group level. By specifically focussing on signal variance that is common to a population, the standard group-analysis GLM approach *obscures meaningful variability at the individual level* (Kanai and Rees, 2011). Yet, quantifying variability in the signal elicited by a given task can be very useful. For our application, a reason to measure the robustness of task BOLD activations is so that meaningful variations at the individual patient level can be appropriately interpreted for surgical planning. TWOM was proposed to specifically evaluate such task activation variability (Seghier and Price, 2016), and was further validated in subsequent work using naming fMRI tasks in healthy controls (Ekert et al., 2021).

**Fig. 3:**
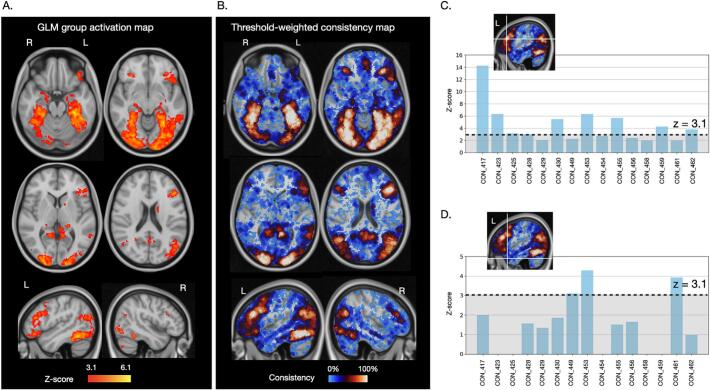
PPTT fMRI task group-average shared activation and inter-individual activation consistency in healthy individuals. PPTT task fMRI results from 15 healthy controls (first visit). A. Results from a standard general linear model group analysis, combining the individual subject task data, identified a left-lateralised fronto-temporal network engaged during semantic processing. Regions with greater between-subject shared variance show higher z-scores (brighter yellow). B. The same individual task activation maps were submitted to an alternate threshold-weighted overlap mapping approach sensitive to inter-individual variation − rather than shared variance − in task activations. Voxels with high activation consistency between individuals are shown in brighter colours (red-to-white); more variably activated voxels in blue. C. An example voxel in left pars triangularis (MNI coordinates −49, 28, 20) that was reliably activated in 15/15 (100%) controls. The z-score threshold delineates the level at which activations were considered significant in single-subject fMRI general linear model analyses. D. In comparison, other regions showed variable consistency; e.g., examining the profile of activations at the single-participant level, the left anterior temporal pole (MNI coordinates −48 18––30) was engaged by only 3 of the controls above significance levels (20%), was activated to some extent by 10 (66.7%) and showed no activation at all in 5 (33%) of the controls. (For interpretation of the references to colour in this figure legend, the reader is referred to the web version of this article.)

For the TWOM approach, the unthresholded z-statistical maps generated in the first-level GLM were registered to standard space and explored at every brain voxel. For every voxel location, a histogram was generated, counting the number of individuals who activated that voxel (y-axis) at a given z-score level (x-axis). Because only areas of task activation are of interest, the minimum z-score was set at 0 and clusters consisting of < 10 voxels were excluded to minimise effects driven by tiny outlier activations. The maximum was defined by the peak z-score detected in the individual subject data. This cumulative histogram (total number of individuals over the range of statistical levels at each voxel location) was converted into a consistency map by calculating the area under the curve of the histogram. Of course, the greater the difference in the observed signal between PPTT trials and line-matching trials, the more likely that the voxel is meaningfully involved in semantic association. Therefore, a weighting factor was applied to allocate more value to higher fMRI signals, as in the original paper (Seghier and Price, 2016). The resulting threshold-weighted overlap maps depict the consistency of individuals activating a given voxel over the specified range of z scores, ranging from 0 (low consistency) to 1 (high consistency). Voxel-specific information can be extracted to visualise the distribution of the task effects across individuals (i.e. which subjects contribute most to the signal at that location, e.g., Fig. 3). Finally, variability in gyral anatomy will naturally result in small discrepancies in like-for-like voxel correspondence between individuals. To minimise the impact of small anatomical variations, as previously recommended (Seghier and Price, 2016), the overlap maps were computed by applying a 1-voxel spherical volume of interest to the inputs (unthresholded contrast of parameter maps). This allowed for sampling of activation information from each voxel as well as its immediately neighbouring voxels, corresponding to a 2 mm radius search sphere for the MNI-2 mm space data. For comparison, larger search radii were explored in the Supplementary Materials.

To avoid searching all voxels in the brain, the original Matlab implementation (Seghier and Price, 2016) used a set of heuristics referred to as ‘relevant voxels’. In the original implementation, relevant subsets were selected by limiting the search to voxels that were activated above set thresholds by at least two subjects, and below ‘extreme’ activation thresholds (equivalent to p < 0.001, uncorrected). The thresholds included activation magnitude (t > 0, in our case z > 0) and cluster size (n > 10 voxels). The rationale for this choice of cluster size was (i) that thresholds on cluster size offer another way to control for false positives (Forman et al., 1995, Woo et al., 2014), (ii) it represents a tiny effect of less than 0.1 cm^3^ (e.g. smaller effects might not be clinically relevant), (iii) smaller clusters than our threshold are likely to show low spatial correspondence across individuals, and (iv) higher thresholds on cluster size might increase the risk of false negatives and might be unnecessary as our approach incorporates a sampling of activation information from each voxel as well as its immediately neighbouring voxels.

Here, we selected similar heuristics in our Python re-implementation (https://github.com/oiwi/twom), with two modifications. Firstly, for sensitivity to possible outlier patient activations, such as due to atypical language organisation, we removed the ‘minimum subjects’ criterion, analysing voxels activated in all individuals. Secondly, because magnitudes of activation may vary between patients and controls, and between acquisition protocols, we analysed all positively task-activated voxels without upper activation limit. For complete flexibility in future use-cases, we also include a new option to search ‘all voxels’ exhaustively (without minimum cluster size). Given a vectorised implementation, these computations take from seconds (‘relevant voxels’) to a few minutes (‘all voxels’) to run on an Apple MacBook M1 Pro laptop. Here, we report findings using our ‘relevant voxels’ implementation.

Consistency of activation can be defined in different ways. We selected voxels engaged by 90 % of the controls in their first visit, and 80 % of the healthy controls across both scan visits, to define brain regions most consistently activated by the task (i.e., showing high inter-individual agreement as well as high test–retest reliability) (see Supplementary Table S2 and Fig. S3). The FSL tool ‘cluster’ was used to extract the MNI coordinates of peak activations from the automatically identified clusters within the overlap maps.

### 2.8 Diffusion MRI tractography

Diffusion data were pre-processed using FMRIB’s Diffusion Toolbox. Typical anatomical distortions in the raw diffusion data were corrected using the tool ‘topup’  (Andersson et al., 2003), which estimates the susceptibility-induced field based on the pair of non-diffusion weighted images acquired in opposite phase encode directions. The resulting estimated fieldmap was input to the tool ‘eddy’ (Andersson and Sotiropoulos, 2016) to average the two phase-reserved datasets and correct for susceptibility artefacts, eddy currents and head motion. Next, a probabilistic diffusion model was fit to the corrected data to derive voxel-wise estimates of fibre orientations and their uncertainties (Jbabdi et al., 2012).

Fibre tracts were generated through probabilistic tractography using a crossing fibre model (Behrens et al., 2007). Connectivity distributions were calculated in each individuals’ diffusion-weighted MRI data from 5 mm spherical regions of interest (ROIs). ROIs were placed at the MNI coordinates of the most consistently task-activated regions, as defined above (Supplementary Fig. S1a). Streamlines were sampled from each voxel in the ROI to the rest of the brain, calculated for every ROI separately. The resulting connectivity distribution maps were normalised to the total number of valid streamlines, and thresholded (at the default 0.001) to remove the least probable streamlines. Tracts from each ROI were summed across individuals for ease of visualisation and anatomical identification. Anatomical labelling of the resulting tracts was done with reference to the FSL XTRACT atlas (Warrington et al., 2020).

### 2.9 Statistical analyses

Post-hoc statistical analyses were performed using SPSS v 29. TWOM was implemented in Python (version 3.8) based on the original Matlab code created for SPM (Seghier and Price, 2016). Non-parametric tests were used to compare task performance accuracy between patient and control groups, and over time in controls. Task activation maps were compared using the Dice similarity index, as implemented in the FSL tool Bianca, computed as: 2 × (volume of overlapping voxels activated during both visits) / (volume of activated voxels during the first visit + volume of activated voxels during the second visit). For Dice comparisons between overlap maps, these were thresholded at 0 (i.e., considering all voxels activated in all subjects). For Dice comparisons of the GLM activation maps, the visit 1 and visit 2 group average maps were thresholded at z = 3.1 (i.e., considering all voxels surviving minimum statistical significance). Findings at p < 0.05 were considered significant. Additional voxel-wise reproducibility and variance analyses are reported in the Supplementary materials.

### 2.10 Neurosurgery

Of the 51 patients included in the fMRI analyses, 32 had a tumour in the language-dominant hemisphere, of whom 18 completed awake intraoperative assessment of language functions. The remaining 13 language-dominant tumour patients declined surgery (n = 1), underwent biopsy only (n = 3) or were operated under general anaesthesia (n = 9, including 6 initially planned for awake surgery). Tasks used to monitor language functions during surgery were selected according to tumour location. Our awake surgery protocol and intraoperative language tasks have been described previously (Voets et al., 2021) and included picture naming, repetition of words or sentences, auditory naming, word reading, sequencing (e.g., days of the week) and, where indicated, picture items of the PPTT. For the present analyses, we focussed on semantic processing errors recorded during either picture naming or the PPTT.

## 3 Results

### 3.1 FMRI individual task data evaluation

The individual task fMRI maps for 3 patients were affected by excessive head motion that could not be adequately corrected by regressing motion parameters. The fMRI data for these 3 patients were therefore excluded from subsequent analyses.

### 3.2 FMRI task performance accuracy

Healthy controls performed the task with high accuracy during both the first (n = 14, mean 94.3 % ± 6.4 %) and second scans (mean 98.3 % ± 2.55 %)(Fig. 2b). Although no performance feedback was given, accuracy improved between the 2 visits (Wilcoxon z = -2.55, p = 0.011). Tumour patients’ accuracy (mean 91.1 % ± 9.3 %) overall did not differ from controls (Mann-Whitney U = 227, p = 0.077). Language dominant hemisphere tumour patients performed marginally worse than controls (n = 30, mean: 91.6 % ± 7.0 %, U = 138, p = 0.07) while those with non-dominant hemisphere tumours performed in the normal range (n = 17, mean: 90.4 % ± 12.6 %, U = 89, p = 0.22). Excluding one outlier (see Fig. 2b), performance of non-language-dominant tumours patients matched that of controls (n = 16, U = 89, p = 0.36).

### 3.3 Standard GLM analyses

The standard group-level GLM analysis of the healthy control first visit data identified *shared* task-related activation of a predominantly left hemisphere fronto-temporal network consisting of 11 regions: 8 cortical, 2 subcortical and 1 cerebellar (Fig. 3a, Table S1). A paired *t*-test GLM analysis comparing the first and second visit healthy control group activation maps did not identify any significant differences over time. Dice overlap between the first visit and second visit GLM group activation maps was 73.3 %.

### 3.4 Threshold-weighted overlap mapping

#### 3.4.1 Cross-subject consistency in healthy controls

To directly probe the consistency of PPTT fMRI activations, we next assessed *variation* in task activations among healthy individuals. Numerous brain regions showed highly consistent engagement during semantic processing (Fig. 3B). Four regions showed overlapping activation in all – or nearly all (>90 %) – controls during their first fMRI scan: the left and right fusiform cortex, left pars triangularis (Fig. 3C) and left superior lateral occipital cortex. Several other regions were more heterogeneously engaged. The left lateral anterior temporal pole, for example, was engaged to some degree by approximately two-thirds of our healthy controls, but to significant levels (z > 3.1) in only 3 individuals (Fig. 3D). Brain regions activated at different consistency thresholds (ranging from 25-100 % of overlap) are reported in the Supplementary Table S2.

#### 3.4.2 Cross-visit reliability in healthy controls

**Fig. 4:**
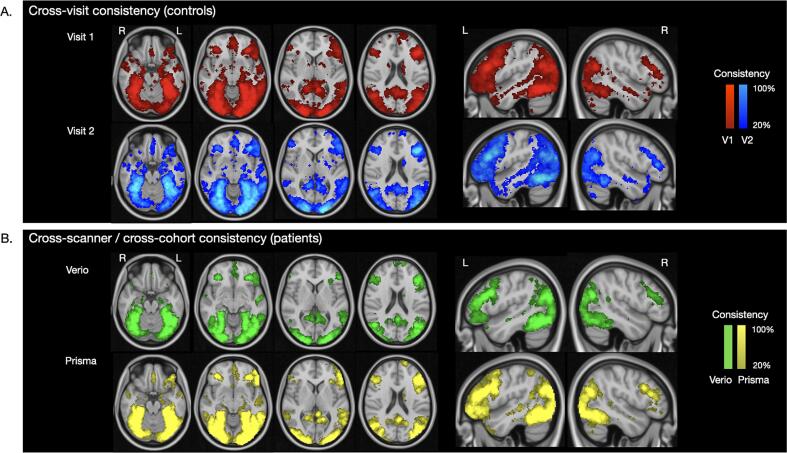
Consistency in activation across visits and scanners. Good reliability was seen for all of our threshold-weighted consistency metrics. A. Test-retest consistency of PPTT fMRI activations among 15 controls scanned twice on the same scanner, at least 3 weeks apart. B. Consistency of PPTT fMRI tasks acquired in 51 patients with a brain tumour, scanned on two different 3 T MRI machines using either a standard clinical fMRI sequence (on the Verio scanner) or an advanced research acquisition (on the Prisma scanner).

Comparing TWOM maps from the first and second fMRI scan in controls, the test–retest consistency of task activations was moderately high (Dice index: 77.2 %). Specifically, the spatial *location* of voxels engaged during semantic processing was largely similar between repeated fMRI scans. Between-visit differences were largely driven by variations in the *extent* of those activations **(**Fig. 4A). Voxels in 9 brain regions were activated in at least 80 % of controls on both visits. These voxels localised to a network of left fronto-temporal and right temporo-occipital regions (Table 2, Fig. S1**)**. Maps at additional consistency levels are illustrated in Supplementary Fig. S3.

**Table 2:** Most reliably task-activated regions based on test–retest analysis.

**Cluster**	**X coordinate (MNI, mm)**	**Y coordinate (MNI, mm)**	**Z coordinate (MNI, mm)**	**Anatomical label**	**Consistency (%)**
1	−28	−66	−12	L occipital fusiform	100
2	30	−70	−10	R occipital fusiform	100
3	−48	27	22	L pars triangularis	100
4	−36	−84	34	L lateral occipital (sup)	93.3
5	−48	−68	22	L lateral occipital (sup)	85.5
6	−10	−54	8	L precuneus	86.7
7	–32	34	−12	L orbitofrontal cortex	89.9
8	48	−64	24	R lateral occipital (sup)	80.1
9	−36	6	32	L inferior frontal sulcus *	80.9

Regions reliably activated during semantic processing in at least 80% of the 15 healthy controls across two separate scan visits, listed in order of descending cluster size. MNI: Montreal Neurological Institute standard brain template. Anatomical labels were derived from the Harvard-Oxford atlas. *The inferior frontal sulcus cluster spanned the junction between the middle frontal gyrus, pars opercularis and precentral gyrus.

#### 3.4.3 Cross-scanner and cross-cohort task fMRI consistency in pre-surgical patients

Finally, we evaluated the consistency of PPTT task activations across two groups of patients scanned on different scanners. Despite the cohort and acquisition parameter differences, there was moderately high similarity (Dice index: 72.8 %) between the TWOM maps obtained from the two patient groups (Fig. 4B). Overall, the spatial distribution of PPTT task activation in patients mirrored that of controls.

**Fig. 5:**
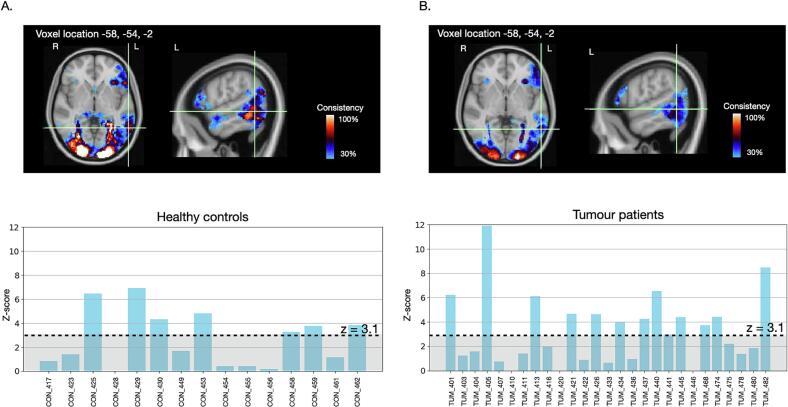
Variability due to consistently weak task activation magnitudes. Several voxels in the left posterior middle temporal gyrus showed heterogeneous PPTT task activation between individuals. Histograms of task activation values (z-score) sampled from a voxel in this location showed that almost all of the 15 healthy controls (A) and 27 patients scanned on the same 3 T Siemens Prisma scanner (B) showed some level of activation during semantic processing. However, the magnitude of activation passed the minimum threshold for statistical significance in approximately half the individuals, and remained below this level for the others. Less robust activation at this location at the population level therefore appeared driven by generally consistent, but variable *magnitudes* in task-activation. Voxel coordinates are in MNI space.

As was also seen in controls, the within-group patient TWOM maps revealed regions that were very reliably activated across patients, and clusters that showed inter-individual variability. The most consistently activated voxels across patients again localised to the fusiform region, with strong fMRI effect sizes during semantic processing observed in nearly every patient (Fig. 4B). The unique added value of overlap mapping is that it enables disambiguation of variation in the activation maps resulting from different scenarios. For example, some voxels may show low consistency because of generally weak activation across individuals. This is illustrated for a voxel in the left posterior middle temporal gyrus in Fig. 5. While this voxel was typically activated to *some degree* during semantic processing by all controls and patients, the level of activation passed the minimum criterion for statistically significant activation in only approximately half the individuals.

**Fig. 6:**
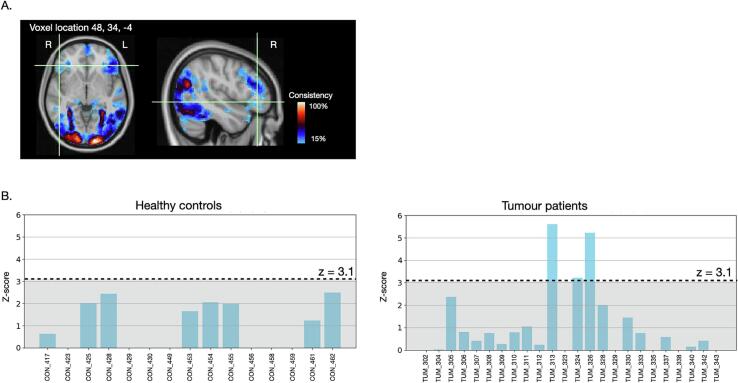
Variability due to individual differences in language processing. A. Several clusters of voxels in the right inferior frontal gyrus showed low PPTT fMRI consistency (activated in 10–20% of tumour patients). B. Plotting the PPTT task activation values (z-scores) from a voxel in right pars triangularis across individuals, 3 / 24 patients scanned on the Verio scanner showed a high level of activation at this location during semantic processing, compared to 0 / 15 controls. In all 3 patients, intraoperative stimulation during surgery for a right hemisphere tumour caused language errors. Variability in activation at this location at the population level therefore appears to reflect meaningful *inter-individual differences* in language processing. Voxel coordinates are in MNI space.

Other brain regions showed variable task activation due to sub-group effects. For example, clusters in the contralateral, right hemisphere inferior frontal gyrus showed low PPTT task activation reliability, indicating high variability at the individual level (Fig. 6A). When plotting the fMRI task effect size from an example voxel, this result was driven by 3 patients (Fig. 6B). Two of these patients (both left-handed) showed very strong activations at this location. Both had a tumour in the right hemisphere, language symptoms at presentation, and had significant right hemispheric language representations based on intra-operative findings. A third patient, also with a right-sided tumour but right-handed, showed a bilateral pattern of fMRI activation and was also found to have right hemispheric language during surgery.

### 3.5 Anatomical pathways underlying the PPTT activated network

Tractography from the 9 most consistently activated ROIs based on test–retest fMRI analysis (i.e., 80 % controls at both visits) revealed a wide network of long-range association tracts (including all major recognised language tracts), commissural and projection fibres. Restricting tractography to the 4 most consistently task-activated regions (90 % of controls) largely replicated this finding. Across all control and patient participants, tractography from these most prominent clusters of activation identified the bilateral optic radiations, inferior longitudinal fasciculus, uncinate fasciculus, ventral occipital fasciculus and inferior frontal-occipital fasciculus, alongside interhemispheric connections along the splenium and genu of the corpus callosum. Additional left-hemispheric streamlines localised to the frontal aslant tract, middle longitudinal fasciculus and superior longitudinal fasciculus complex (branches I, II, III, arcuate fasciculus) (Supplementary Fig. S1B &C).

### 3.6 Intra-operative direct brain stimulation

Among the 18 patients who underwent awake intraoperative assessment of language functions, and excluding ‘no response’ errors (anomia), clear and reproducible semantic paraphasias were elicited in 8 patients. The corresponding sites were cortical in 1 patient and subcortical in 7 patients. The cortical stimulation error was encountered at the mid portion of the middle temporal gyrus / superior temporal sulcus. The subcortical errors localised to the patient’s tractography-predicted IFOF in 6 cases, and occurred in the peritrigonal region (i.e., likely IFOF) in 1 case. Example cases are illustrated in Supplementary Fig. S4.

## 4 Discussion

We implemented and evaluated a fMRI version of the Pyramids and Palm Trees Task (PPTT), a semantic association task commonly used in clinical practice. Using a previously validated threshold-free overlap mapping (TWOM) approach, we found good test–retest and between-subject reliability of PPTT activation maps in healthy controls. Comparable consistency was seen in two cohorts of presurgical glioma patients scanned on different MRI scanners. The PPTT activated distributed language networks, including – but not limited to – brain regions commonly implicated in semantic processing. By combining the power of group studies with sensitivity to inter-individual variability, TWOM additionally spotlighted subgroup effects, including activations driven by atypical language organisation. We propose TWOM as a simple but powerful approach for (1) research evaluation of clinical fMRI tasks and (2) uncovering patterns of brain activation reflecting meaningful differences in brain function.

### 4.1 Implementation of the PPTT for presurgical fMRI

Many previously “inoperable” gliomas can now be safely resected using awake intraoperative language mapping (Benzagmout et al., 2007, Southwell et al., 2017). Nevertheless, persisting language declines affect ∼14.6 % of language-dominant hemisphere surgeries (Collée et al., 2022), ranging up to 25 % for tumours in high-risk (Gupta et al., 2007, Southwell et al., 2017) regions. These deficits are commonly attributed to surgical complications or ‘failed’ awake surgery due to seizures (Morshed et al., 2021) or patient non-cooperation (Nossek et al., 2013). However, the challenging environment and time constraints of awake ESM place practical limits on exhaustive language mapping. Limited functional testing can, therefore, also engender new language deficits through incomplete or ‘false negative’ ESM findings (Mandonnet et al., 2010). Consequently, advance knowledge of language network organisation from fMRI provides useful complementary information, for example to tailor ESM. However, frequently, the language tasks performed during fMRI differ from those used in the neurosurgical setting (Weng et al., 2018, Holloway et al., 2022), limiting inferences that can be drawn from pre- to intra-operative findings.

Historically, the focus for surgical language mapping was to preserve speech through visual object naming (Penfield and Roberts, 1959). With longer glioma survival rates, there is a growing focus on preserving broader skills (Duffau, 2021). A challenge to mapping complex cognitive functions is our incomplete understanding of how these are represented in the brain. One example is semantic processing. ESM can selectively disrupt lexico-semantic access or higher-level semantic processing (Duffau et al., 2005, Gatignol et al., 2004, Moritz-Gasser et al., 2013, Corrivetti et al., 2019). Hence, it has become common to assess semantic performance intra-operatively using picture naming or, increasingly, the picture variant of the PPTT (Rofes et al., 2017, de Zwart and Ruis, 2024). In contrast, most pre-operative semantic processing fMRI tasks involve decisions on word categories (e.g., (Binder et al., 1997)) or word pair relatedness (e.g., (Seghier et al., 2004). Our aim here was not to establish the ‘best’ task to map semantic processing. Rather, we adapted a pictorial PPTT from neuroscience studies (Ricci et al., 1999, Vandenberghe et al., 1996, Baciu et al., 2016, Josse et al., 2008) – permitting assessment of semantic knowledge even in patients with verbal (phonological / orthographic) deficits – to pragmatically facilitate alignment with neurosurgical practice.

### 4.2 Task feasibility

Most participants performed the PPTT task with high accuracy. One patient with a left fronto-temporo-insular glioma had substantial cognitive difficulties at the time of fMRI and could not follow the button-box response instructions. Others found some out-dated stimuli obscure, highlighting known challenges with the original PPTT (Klein and Buchanan, 2009). Similarly, during awake surgery, the higher demands of the PPTT affected compliance, resulting in use of simpler picture naming task in many cases. Other groups have described similar challenges, reporting a higher percentage of patients unable to complete the PPTT in surgical settings (13.6 %) compared to those unable to perform picture naming (3.8 %) (Albuquerque et al., 2024). Therefore, while PPTT performance was practically feasible most of the time, updated stimuli – or overall simpler tasks – might prove optimal to monitor semantic processing across the wider range of glioma patients.

### 4.3 Global consistency of fMRI task activations

At the group level, semantic decisions activated a distributed, left hemisphere fronto-temporo-occipital network in controls and glioma patients. These results align with previous neuroimaging findings using comparable tasks in healthy and neurological populations (Ricci et al., 1999, Seghier et al., 2011, Baciu et al., 2016, Vandenbulcke et al., 2006, Nelissen et al., 2011, Chee, 2006, Josse et al., 2008, Vandenberghe et al., 1996, Vandenberghe et al., 2013). Furthermore, in addition to the high Dice overlap between scan visits, direct comparison of activation maps across scan visits in controls found no significant differences using standard GLM analyses, replicating one prior test–retest study using the written word PPTT (Chee et al., 2003). That earlier study noted stabler single-subject inter-session activations at higher statistical thresholds. The critical influence of statistical thresholding on fMRI, especially in surgical populations, is well recognised (see (Voets et al., 2025a), and threshold-weighted or threshold-free approaches are universally recommended (Vingerhoets et al., 2023). Contributing reasons are that the fMRI blood oxygen level dependent signal is affected by various technical, physiological, clinical and cognitive factors (e.g., medications, pathology, effort) (Haller and Bartsch, 2009). Amplitudes of task-evoked fMRI are therefore frequently reduced in elderly and patient groups (Haller and Bartsch, 2009, D’Esposito et al., 2003), complicating the interpretation of analyses based on fixed statistical cut-offs. TWOM offers a complementary approach by establishing consistency across a range of activation levels, derived directly from the data (Seghier and Price, 2016). Comparing both analytical approaches, the most reliably activated brain regions identified by TWOM coincided with those found using the standard GLM (see also Supplementary Fig. S5). Taken together, our findings highlight robust PPTT task-related activation across subjects, visits, cohorts and scanners, supporting theoretical suitability for clinical use. Of note, there was no statistical difference in the task activation magnitude as a function of acquisition protocol (clinical vs research-grade). However, the range and associated variance was high between patients in both cohorts, likely obscuring a group-average benefit of accelerated sequences observed in previous studies (e.g. (Voets et al., 2021)).

### 4.4 Drivers of regional fMRI variability

A growing body of work indicates that individuals’ brain activity co-varies with behavioural traits (see (Finn, 2021)), symptoms (e.g., pain response (Kohoutová et al., 2022)) and clinical outcomes (e.g. motor recovery post-stroke (Favre et al., 2014)). These observations motivate growing efforts to characterise variability in neural processing at the individual level (Seghier and Price, 2016, Voets et al., 2019). Beyond the core task-activated regions, TWOM identified areas of high inter-subject variability not apparent from the group GLM results. These regionally heterogeneous PPTT fMRI activations may have several causes.

Firstly, although no feedback was given, controls showed a small but significant performance improvement between visits, as observed previously (Klein and Buchanan, 2009). Test-retest instability did not, however, explain high signal variability in regions such as the left anterior temporal lobe (ATL): the 3 controls who activated the lateral ATL did so consistently between visits, while the 5 controls without ATL engagement showed no activation on either visit.

An alternative explanation is the availability of different neural pathways to perform a given function, i.e., ‘degeneracy’ (Price and Friston, 2002). Previous research revealed different strategies – and underlying neural routes – for language processes such as reading (Seghier et al., 2012) and repetition (Sajid et al., 2023). These alternate pathways explain why the same lesion may spare functions in one patient but impair others (Seghier et al., 2012). Further evidence is, of course, needed. However, our observed sensitivity to patient subgroups showcases the potential of TWOM to identify candidate alternate neural routes supporting complex semantic processing across the population.

Of direct clinical interest, overlap mapping disambiguated variability driven by (a) *weak* activation seen *consistently* across participants from (b) *strong* activation in just a *few individuals*. The latter ‘outlier effects’ are especially relevant in neurosurgical populations if these variations at the neural level relate directly to behaviour. Plotting which individuals contributed to regions of variable task activation highlighted 3 patients in whom right inferior frontal engagement deviated notably from other patients and controls. Awake surgery with ESM confirmed the essential contribution of the right hemisphere to language in these cases. This result highlights a unique advantage of TWOM. By not depending on standard metrics of variance in fMRI, TWOM depicts typical activation patterns at the group level without obscuring individual variability. In this way, TWOM offers a window onto variability in fMRI activation patterns that can be directly probed to explore their sources and behavioural validity. In this initial study, we included a clinically representative but varied range of patients (including left and right-hemisphere language dominance and tumours in different locations). Consequently, more in-depth subgroup analyses could not be meaningfully conducted, and further exploration in larger, more homogeneous cohorts is needed. Additionally, and importantly, standard BOLD-fMRI makes numerous assumptions about haemodynamic responsiveness. In the presence of pathology, these assumptions may not be met. For example, gliomas at different stages show vascular proliferation and disruption to the blood brain barrier among other pathological features that may affect neurovascular coupling (Cuddapah et al., 2014). Separate from variations in neural network organisation, neurovascular uncoupling is therefore a potential alternate source of variability in task activations between patients.

### 4.5 Aligning fMRI and surgical practices

Semantic processing errors represent a low percentage (∼14 %) (Collée et al., 2023) of all intraoperative language errors, but are reported in a wide range of cortical locations (including superior temporal, orbitofrontal, inferior parietal and dorsolateral prefrontal) (Duffau et al., 2005, Gatignol et al., 2004, Moritz-Gasser et al., 2013, Corrivetti et al., 2019, Corina et al., 2010). Most semantic processing errors, however, occur in the deep white matter (Collée et al., 2023, Albuquerque et al., 2024). Specifically, ESM implicates the language-dominant hemisphere IFOF in verbal semantic processing (Cocquyt et al., 2020, Collée et al., 2023). Concordantly, in our patients, most (8 / 9) intra-operative semantic processing errors were encountered in proximity to the IFOF. It has been proposed that the IFOF serves as a “direct” route for semantic processing, with additional tracts (uncinate, inferior longitudinal fasciculus) providing “indirect” routes (Moritz-Gasser et al., 2013). Alternatively, different fibre tracts (and the cortical regions they connect) may support different *types* or *aspects* of semantic processing. A proposed example includes the likely differential roles of the medial vs lateral ATL for object vs verbal stimuli (Rice et al., 2015). Currently, the neural representations of sematic processing remain a topic of active research, and may be heterogeneous (Frisby et al., 2023). Therefore, ESM applied to each of the task-activated brain regions might elicit distinct semantic error profiles. Only one patient in our limited series exhibited semantic paraphasias during stimulation of the cortex, at the level of the middle temporal gyrus. This area has been implicated more in ‘thematic’ than ‘taxonomic’ relationships (Zhang et al., 2023). However, due to exposing only the minimum cortex needed for surgical access, and due to the rarity of intraoperative semantic errors, our present data cannot further elucidate behavioural specialisations within the wider task-activated network. Diffusion tractography from the 4 most consistently task-activated clusters, however, identified all long-range association tracts, alongside commissural fibres. Therefore, the PPTT task, at least in our implementation, appears to offer very high *sensitivity* to language networks, at a cost of *limited specificity* to regions specifically associated with semantic processing.

### 4.6 Limitations

Several limitations are important to consider. For task simplicity, we selected non-nameable line drawings as a control condition. This approach is akin to common-place use of non-linguistic stimuli in word decision tasks (Binder et al., 1997) and scrambled images in picture-matching tasks (Rice et al., 2018), but may accentuate implicit naming during ‘semantic’ trials. Alternative task contrasts (e.g. matching pictures by size) may offer greater specificity for semantic processing. Additionally, our sample sizes were limited for practical reasons. While TWOM readily identified single individuals with atypical language, demonstrating clinical applicability, our effective sample size to detect a wide range of subgroup effects was limited. Further subgroup effects – such as relevant to degeneracy in semantic processing – may be uncovered in larger cohorts. Regarding intraoperative confirmation, not all patients were operated awake and the areas exposed in awake surgeries varied. Cortical ESM-induced semantic paraphasias are reportedly rare, but our ability to interpret cortical fMRI activations through stimulation interference was particularly limited in our cohort. Additionally, we did not acquire a full set of diagnostic sequences required for accurate tumour characterisation and quantification. This reflected practical limitations with administering contrast agents in a purely research setting, and subsequent choice to complement rather than duplicate existing recent clinical radiological information. We therefore only defined tumour masks on the research T1-weighted scans − on which most registration tools rely – in order to drive registration based on ‘normal appearing’ voxels as per standard practice. The T1-derived tumour masks were visually verified by a consultant neuroradiologist with reference to patients’ recent clinical diagnostic imaging.

## 5 Conclusions

Mapping and preserving semantic processing in surgical populations raises numerous practical and conceptual challenges. We show how a simple analysis approach (TWOM) can be applied to assess the reliability of fMRI activation maps, illustrated here using a single task chosen to closely match intraoperative practices. Relative to standard group-analysis approaches, TWOM offers insight into both ‘typical’ task activation patterns as well as regionally heterogeneous activations driven by individual variability. The latter offers direct insight into neurosurgically-relevant variants in neural processing of direct relevance to behaviour.

## CRediT authorship contribution statement

**Natalie L. Voets:** Writing – review & editing, Writing – original draft, Visualization, Validation, Resources, Project administration, Methodology, Investigation, Funding acquisition, Formal analysis, Data curation, Conceptualization. **Oiwi Parker Jones:** Writing – review & editing, Visualization, Software, Methodology, Investigation, Formal analysis, Conceptualization. **Mohamed L. Seghier:** Writing – review & editing, Software, Resources, Methodology. **Puneet Plaha:** Writing – review & editing, Validation, Funding acquisition.

## Declaration of competing interest

The authors declare that they have no known competing financial interests or personal relationships that could have appeared to influence the work reported in this paper.

## Data Availability

Data are available from the corresponding author upon reasonable request, subject to ethical approvals and institutional data sharing agreements. Threshold-free overlap mapping Python scripts are freely available from https://github.com/oiwi/twom.
