# Supplementary material for: Separating the forest from the palm trees: Individual variation in a presurgical language mapping task
Source: Neuroimage Clin. 2026 Jan 5;49:103943. doi: 10.1016/j.nicl.2026.103943 (PMC12816848; doi:10.1016/j.nicl.2026.103943)
Supplement: Supplementary Data 1 [file mmc1.docx]

**Supplementary Materials accompanying Voets et al., (“Separating the forest from the palm trees: Individual variation in a presurgical language mapping task”).**

Methods

*Voxel-wise reproducibility and variance comparisons between GLM and TWOM*

Group-level GLM and TWOM spatial maps capture distinct aspects of individual subject task-activation maps. The former (GLM) represents areas activated in common across the individuals within the cohort, while the latter (TWOM) specifically aims to represent individual variability in activation within the overall cohort. By directly comparing the two approaches, we would expect to observe areas of both high correspondence and areas of divergence between the methods. We, therefore, explored 3 metrics to express voxel-wise reproducibility and variance between the GLM and TWOM approaches. For the control group (n = 15) and separately for one of the patient cohorts (Verio patients, n = 24), we calculated:

a) Pearson’s correlations between the GLM group activation maps and the TWOM consistency map (at a range of consistency levels from 0.0 to 0.9), computed across all brain voxels.

b) coefficients of variation (CV) computed across individual (i.e. first-level GLM-derived) task activation maps. We concatenated the individual subject task activation maps and computed voxel-wise standard deviation across time, separately for controls and for patients. CV was then expressed as CV = SD / |mean|, stabilised with epsilon (1e-6). Inspection of the resulting CV spatial map revealed that voxels fell into two classes, those with low CV values (range 0-1) and high CV values (>1). Since there should be smaller coefficients of variation in voxels that are more consistency activated, the count of ‘low CV’ voxels should increase with higher levels of consistency. To plot histograms representing the distribution of CV at different levels of spatial correspondence, we extracted the count of voxels in the ‘low CV’ and ‘high CV’ ranges, within the TWOM map, thresholded at different levels of activation consistency.

c) leave-one-out similarity analyses. To directly quantify voxel-wise reproducibility between TWOM and GLM approaches, we performed leave-one-out (LOO) analyses to derive a series of GLM maps (representing group activation for n - 1) and a series of TWOM maps (representing consistency of activation for n - 1). We then calculated pair-wise similarities between LOO maps (Pearson’s correlations) providing a distribution of LOO similarities for GLM, and separately a distribution of LOO similarities for TWOM. Using permutation testing, we directly compared the GLM and TWOM distributions to test for any difference in mean reproducibility between methods.

*Defining regions of interest for tractography*

Connectivity distributions were calculated from 5mm sphere masks placed on the MNI coordinates of the most consistently task-activated brain regions. We used the test-retest activation results in healthy controls to define these regions, based on the premise that the most reliably engaged brain regions during task performance should be highly reproducible between scan visits. We therefore masked the unthresholded overlap maps derived from the first visit with the same maps calculated on the second visit, to retain only those brain regions activated in both scans. Next, we thresholded the overlap maps at values ranging from 25% to 90% of all voxels activated by all controls in both visits (Fig. S1).

# **Figure S1. Tractography from the most consistently task-activated clusters.**


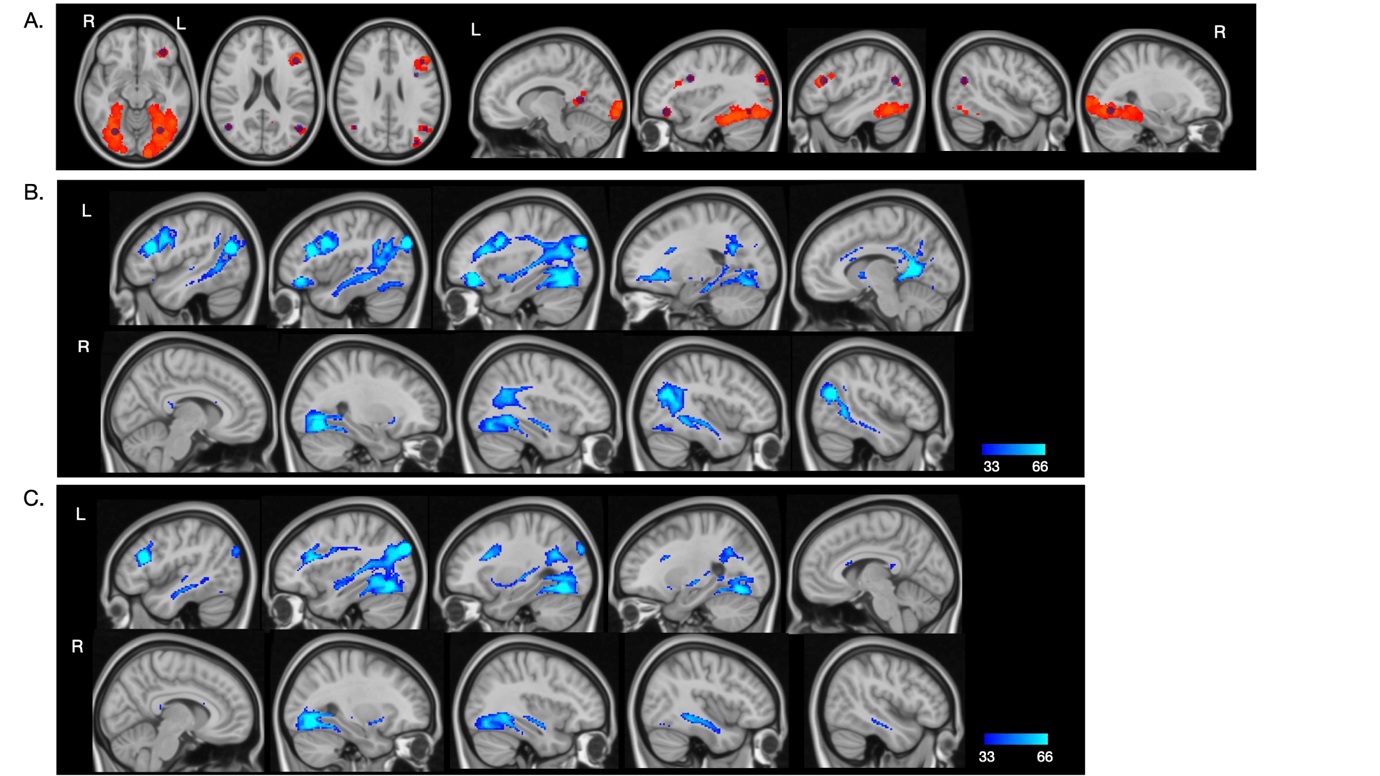


*Legend*. A. Brain regions consistently engaged during PPTT-fMRI (red) based on test-retest results in healthy controls (voxels activated in 80% of the controls at both visits). The coordinates of the centre of gravity of the activated clusters were extracted and a 5mm spherical regions of interest created at this location (blue circles) for tractography. B. Diffusion tractography in all healthy controls and brain tumour patients, sampled from the 9 most consistently activated clusters during semantic processing based on test-retest analysis in controls, and the top 4 most consistently task-activated clusters (90% of controls) (C). Streamlines were summed together across individuals and thresholded to show tracts common to half the population for ease of visualisation. All of the major long-range association fibre tracts involved in language processing were identified.

Visual inspection of the resulting thresholded overlap maps indicated the task-activated brain regions to be very stable in the 75-85% range. We therefore selected the 80% consistency threshold to define the most reliably activated PPTT-task regions. Using the FSL tool cluster, we automatically extracted the MNI coordinates of the centre of gravity of all clusters detected in this consistency map at this threshold. The resulting 9 supratentorial clusters (Main paper, Table 2) were used to define regions for tractography.

Results

*Effect of increasing TWOM search radii*

The consistency maps generated using the default 1 voxel search radius were spatially comparable to maps generated using a wider search radius (2 or 3 voxels). At the centre of activating clusters, these maps yielded similar results, while larger search kernels showed expected edge or smoothing effects (i.e., a larger proportion of controls reported as activating voxels at the edge of clusters when using larger search kernels (Fig. S2).

# **Figure S2. Effect of search radius on consistency results**


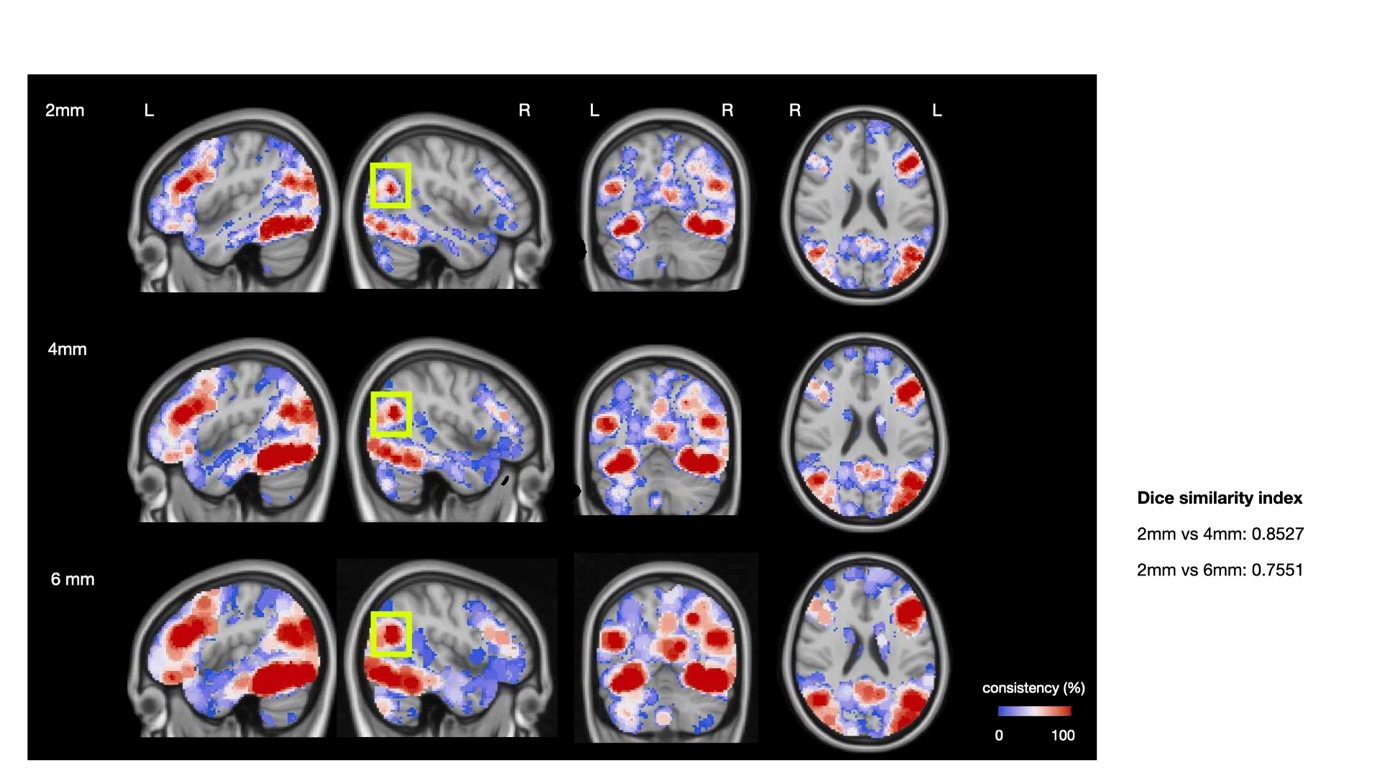


*Legend*. Threshold-weighted overlap maps in healthy controls, generated using the default search radius (1 voxel, corresponding to 2mm) and larger radii (2 voxels = 4mm, 3 voxels = 6mm). Different search kernels yielded maps that were spatially similar. Increasing the radius created expected effects, resulting in larger numbers of participants activating voxels at the edge of a cluster (illustrated in highlighted box).

# **Table S1. GLM results controls v1 coordinates for Z-max activations**

| **Hemisphere** | **Z-max** | **Coordinates** | **Anatomical region** |
| --- | --- | --- | --- |
| Left | 5.14 | -38, 36, -18 | Orbitofrontal cortex |
|  | 4.61 | -6, 22, 48 | Paracingulate / Superior frontal gyrus |
|  | 4.03 | -28, -60, 46 | Lateral occipital cortex (superior) |
|  | 4.26 | -14, 2, 16 | Caudate nucleus |
|  | 4.11 | -26, 24, 56 | Superior frontal gyrus |
| Right | *6.11* | *28, -44, -12* | Temporo-occipital fusiform |
|  | 4.42 | 22, -2, -14 | Amygdala |
|  | 4.38 | 6, -48, -46 | Brainstem / cerebellum |
|  | 4.34 | 30, 32, -8 | Orbitofrontal cortex |
|  | 4.29 | 44, 20, 28 | Inferior frontal sulcus / middle frontal gyrus |
|  | *4.18* | *50, -64, 24* | Lateral occipital cortex (superior) |

*Legend*. Regions of maximum task-activation during semantic processing (task contrast semantic > lines) from a standard general linear model group analysis of the first visit fMRI in 15 healthy volunteers. Coordinates are reported in MNI space.

# **Table S2. Consistency of activations at various ranges of population overlap**

| **Consistency** | **Anatomical label*** | **Coordinates** | **Anatomical label** | **Coordinates** |
| --- | --- | --- | --- | --- |
| **90%** | **Left hemisphere** | | **Right hemisphere** | |
|  | Occipital fusiform | -30 -70 -12 | Occipital fusiform | 30 -72 -10 |
|  | Pars triangularis | -46 28 22 |  |  |
|  | Sup. lateral occipital cortex | -36 -84 34 |  |  |
| **75%** | **Left hemisphere** | | **Right hemisphere** | |
|  | Occipital fusiform | -30 -70 -12 | Occipital fusiform | 30 -70 -10 |
|  | Lateral occipital (sup.)(lateral cluster) | -42 -76 -26 | Lateral occipital (sup.) | 46 -64 22 |
|  | Inferior frontal sulcus / Middle Frontal Gyrus | -42 22 24 | Cerebellum | 34 -70 -46 |
|  | Precuneus | -8 -54 8 |  |  |
|  | Orbitofrontal cortex | -36 36 -12 |  |  |
|  | Lateral occipital (sup.) | -26 -72 44 |  |  |
| **50%** | **Left hemisphere** | | **Right hemisphere** | |
|  | Lingual gyrus | -8 -68 0 | Cerebellum | 34 -72 -46 |
|  | Pars opercularis | -42 22 22 | Inferior frontal sulcus / Middle Frontal Gyrus | 46 26 22 |
|  | Medial sup. frontal gyrus | -4 30 48 | Orbitofrontal cortex | 34 36 -12 |
|  | Medial frontal cortex | -4 44 -12 | Cerebellum / brainstem | 4 -54 -48 |
|  | Amygdala | -18 -4 -16 | Pars triangularis | 48 40 8 |
|  |  |  | Amygdala | 20 0 -18 |
| **25%** | **Left hemisphere** | | **Right hemisphere** | |
|  | Cuneus | -12 -46 6 | Pars triangularis | 44 28 12 |
|  | Medial frontal cortex | -2 44 -12 | Cerebellum / brainstem | 4 -48 -46 |
|  | Thalamus | -12 -4 14 | Temporal pole | 42 22 -30 |
|  | Parahippocampal gyrus | -22 0 -42 | Caudate | 14 2 12 |
|  | Supramarginal gyrus | -62 -28 34 |  |  |
|  | Postcentral gyrus | -50 -18 38 |  |  |
|  | Temporal pole | -46 17 -31 |  |  |

*Legend*. Anatomical labels and centre of gravity MNI coordinates for overlap maps at different consistency thresholds (i.e., % population overlap). Sup. = Superior.

# **Fig S3. PPTT task fMRI test-retest maps at various** **consistency thresholds**


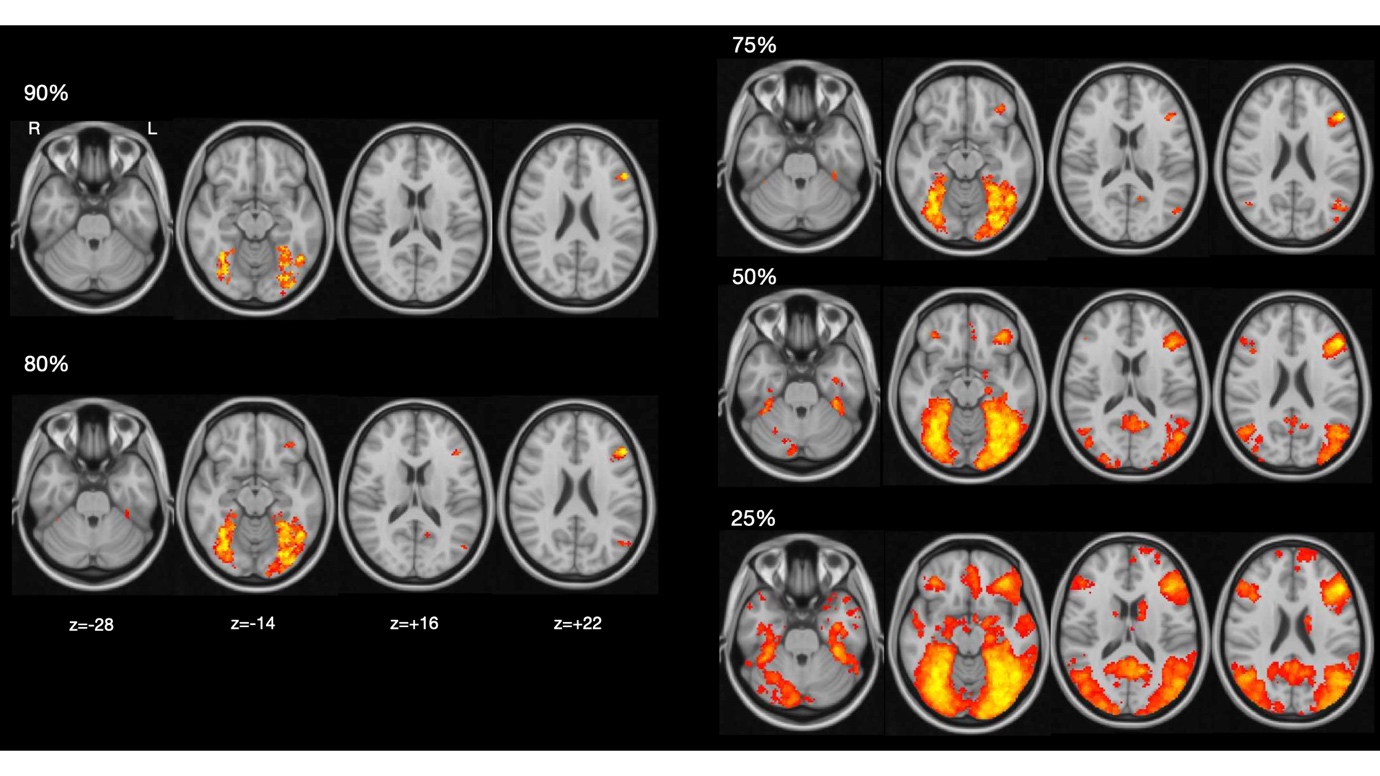


*Legend*. Consistency of activations in 15 healthy controls, scanned on two separate occasions at least 3 weeks apart. The visit 1 consistency map was masked by the visit 2 consistency map and thresholded at different levels of overlap (corresponding to percentage of the controls activating a given threshold) to derive ‘most reliably’ task-activated brain regions across both visits. See Table 2 (main text) and Table S2 for corresponding anatomical labels and voxel coordinates.

*Voxel-wise reproducibility and variance comparisons between GLM and TWOM*

1. Voxel-wise Pearson’s correlations performed across the GLM group activation map and TWOM maps (at a range of consistencies) in controls and in patients showed high correlations between both approaches at most thresholds (Table S3, Fig S5A). At very high levels of ‘thresholding’ of the TWOM maps, the correlation with GLM declined, likely due to the reducing number of voxels at very high consistency levels.

| TWOM threshold | Controls – Pearson’s R | Verio patients – Pearson’s R |
| --- | --- | --- |
| 0.0 | 0.6739 | 0.7737 |
| 0.1 | 0.6662 | 0.7676 |
| 0.2 | 0.6707 | 0.7977 |
| 0.3 | 0.6998 | 0.8202 |
| 0.4 | 0.7238 | 0.7847 |
| 0.5 | 0.7479 | 0.6865 |
| 0.6 | 0.7455 | 0.5145 |
| 0.7 | 0.7017 | 0.3088 |
| 0.8 | 0.5990 | 0.1271 |
| 0.9 | 0.3748 | 0.0253 |

1. Voxels with low coefficients of variation corresponded spatially to regions of high TWOM consistency and, as expected, the distribution of ‘low CV’ voxels (derived from the GLM maps) increased at higher levels of TWOM consistency (Fig. S5B).
2. Both for controls (mean difference GLM – TWOM : -0.0749, p < 0.001) and for patients (mean difference GLM – TWOM : -0.036, p < 0.001), voxel-wise reproducibility was higher for TWOM LOO than for GLM LOO (Fig. S5C).

# **Figure S4. Illustrated sites of semantic errors during awake surgery**


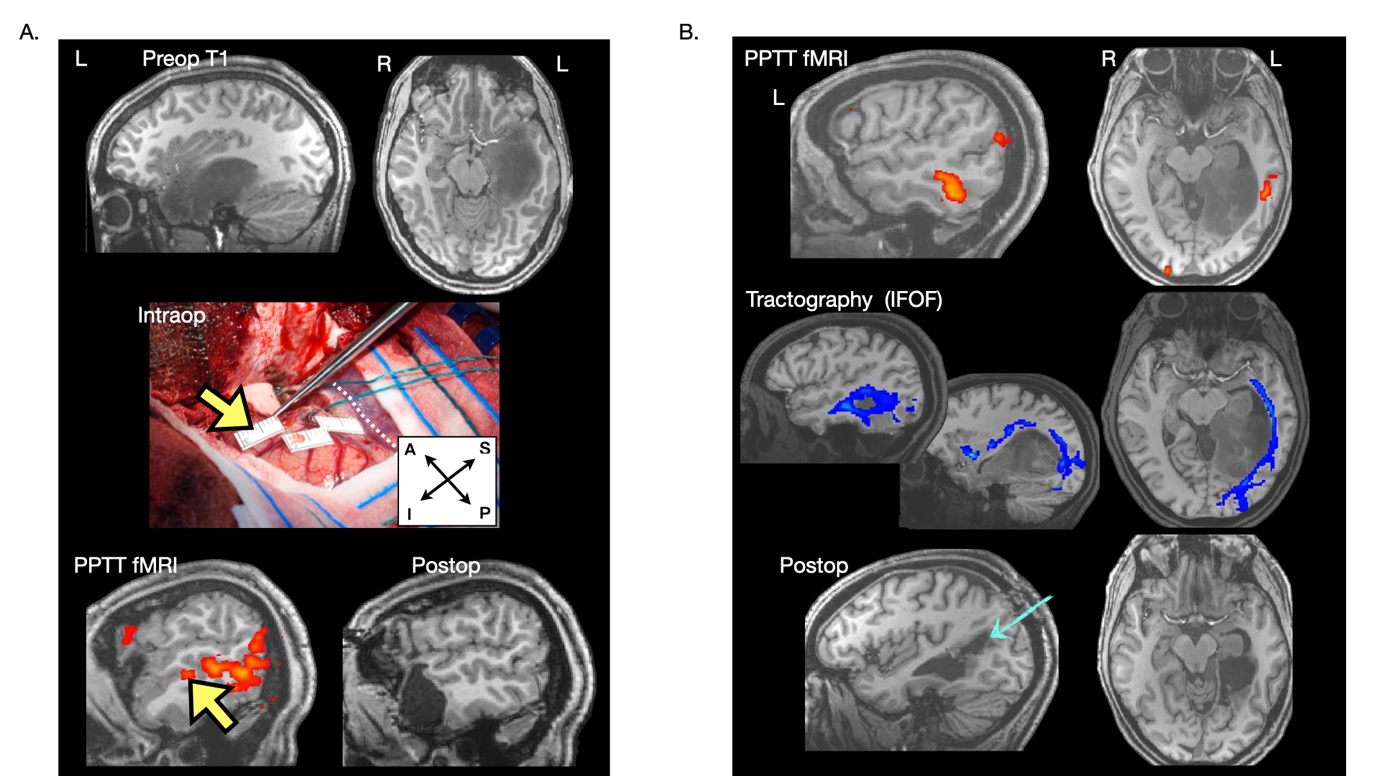


*Legend*. Example patients who experienced semantic paraphasias during intraoperative brain stimulation. A. This 24-year-old right-handed man was admitted to hospital acutely unwell with fever, hallucinations and partial seizures. MRI indicated a non-enhancing glioma in the left anterior medial temporal lobe. Pre-operative fMRI indicated left hemisphere dominance for language. Cortical stimulation at 5mA identified repeated semantic paraphasias at the posterior margins of the intended corticotomy (middle image, yellow arrow) corresponding to the middle temporal gyrus activated during PPTT-fMRI (lower row, activations in red). The white dotted line indicates the Sylvian fissure. B. This 55-year-old man presented with visual symptoms and seizures. Preoperative imaging indicated a left posterior medial temporal lobe glioma and left hemisphere dominance for language. The surgical trajectory was revised to access this tumour from a supero-posterior approach, based on fMRI and tractography results indicating language and optic tracts lateral to the tumour. Subcortical stimulation at 6mA induced reproducible anomia and multiple semantic errors (semantic paraphasias and sudden inability to recognise the meaning of the PPTT stimuli) at the location of the inferior fronto-occipital fasciculus (IFOF). PPTT: Pyramids and Palm Trees Test.

# **Figure S5. Voxel-wise reproducibility and variance comparisons between GLM and TWOM.**


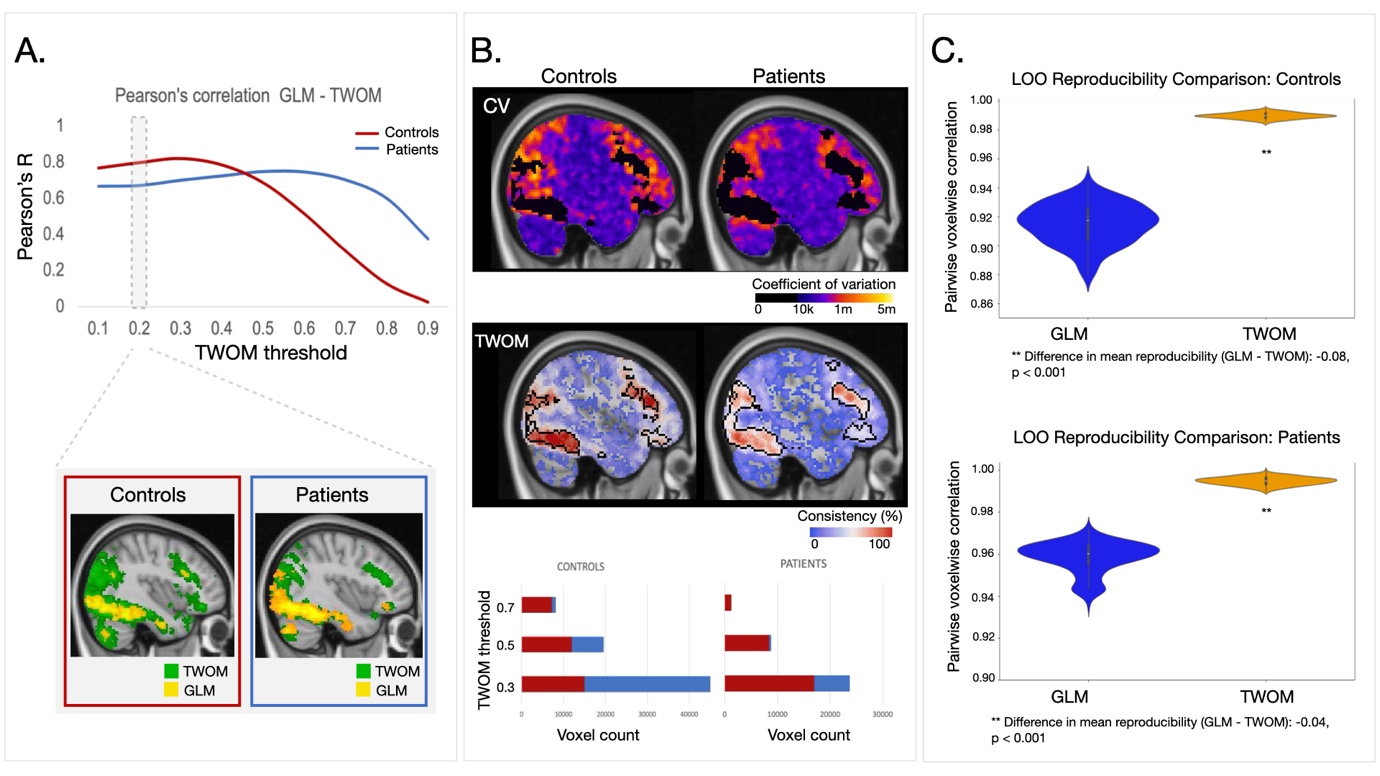


*Legend*. A. Results of voxel-wise Pearson’s correlations between the group GLM activation map and group-level TWOM map calculated separately for the controls and one of the patient cohorts, showing high correlations between methods at most TWOM levels. B. Spatial map highlighting voxels with high (purple-red-yellow) or low (black) coefficients of variation, calculated across the individuals in the control and patient cohorts. Voxels with low GLM-derived coefficients of variation co-localised to areas of high TWOM consistency. At higher levels of TWOM consistency, only voxels showing very low coefficients of variation were found. C. Violin plots showing the distribution of voxel-wise reproducibility from GLM-LOO and TWOM-LOO. Direct comparison of the distributions of the GLM and TWOM approaches revealed significantly higher voxel-wise reproducibility for TWOM-LOO than for GLM-LOO (both p < 0.001). GLM = general linear model. TWOM = threshold-weighted overlap map. LOO = leave-one-out (analysis).
